# A Breath of Fresh Air: Carbon Emissions From Inhaler Devices and the Role of Prescribing Clinicians in Reducing Their Environmental Harm

**DOI:** 10.7759/cureus.106990

**Published:** 2026-04-13

**Authors:** Jahin I Ahmed

**Affiliations:** 1 General Medicine, Goulburn Valley Health, Shepparton, AUS

**Keywords:** asthma, carbon emissions, environmental harm, inhaler devices, prescribing

## Abstract

The use of inhaler devices, particularly pressurised metered dose inhalers (pMDIs), has been a particular concern given their contribution to carbon emissions. One such method is through the release of propellant gas composed of hydrofluorocarbons (HFCs). Various methods, such as changing HFC types and implementing recycling programs, aim to reduce carbon emissions. As clinicians, we can change our prescribing behaviour, such as prescribing soft mist inhalers (SMIs) and dry powdered inhalers (DPI), alongside regular review, to ensure our actions play a role in minimising carbon emissions from inhaler device use.

## Editorial

The causal relationship of carbon emissions leading to the subsequent contribution to climate change and global healthcare outcomes is well established [1]. Healthcare itself plays a large role in the contribution of carbon emissions, making up approximately 7% of the total carbon emissions from all causes in Australia [2]. From this 7%, approximately 13% of the emissions are directly related to the use of asthma inhaler devices, including but not limited to pressurised metered dose inhalers (pMDI), dry powdered inhalers (DPI), and soft mist inhalers (SMI). The main sources of carbon emissions emanate from the various stages of the inhaler life cycle, including raw materials, production, distribution, use of inhalers, and their disposal [3]. As clinicians, we must appreciate the climate effects of inhaler devices, given their widespread use for epidemic respiratory conditions such as asthma and chronic obstructive pulmonary disease (COPD). However, we must also not compromise on quality evidence-based medicine, which would impact patient morbidity and mortality. It is striking the balance between these two conflicts that clinicians must achieve to ensure that the clinician acts in a socially responsible manner whilst providing quality health care.

Sources of carbon emissions

A large proportion of carbon emissions from asthma inhaler devices (pMDIs) are derived from the propellants used to aerosolise the medication for rapid absorption. An estimated 0.03% of all total greenhouse gas emissions were directly linked to propellant use in 2018 [4]. The initial propellants used were based on a hydrocarbon called chlorofluorocarbons (CFCs); however, given the malevolent contributions of CFCs to ozone depletion, the propellant was phased out, and hydrofluorocarbons (HFCs) were introduced as an alternative after the Montreal Agreement (1987). Though it proved to be an improvement from its predecessor due to HFC not having effects on the ozone layer, it still possessed the ability to contribute to carbon emissions, given its long half-life and heat-trapping ability, which is notably more potent than CO2 [3].

The severity of HFC carbon emissions can be measured through a calculated index system called the Global Warming Potential over 100 years (GWP-100), which measures the heat-trapping effects of various greenhouse gases relative to CO2. The GWP of HFC compared to CO2 has been noted to be in the thousands, with maximum ranges from 9900 to 36500; however, this is dependent upon the subtype of HFC propellants used. In current inhaler devices, the propellant HFC 134a, which is commonly used in salbutamol, has a GWP of 1530, while HFC 227ea, which is found in preventer budesonide/formoterol, has a GWP of 3600. In 2022 alone, there was a total of 17,307 kg of CO2 equivalent (kgCo2e) emission from propellants alone, of which 13,572 kgCo2e came from HFC 134a and 3,735kg from HFC 227ea [3,4].

Given the vast quantity of HFC released into the atmosphere and its potency compared to CO2, it poses a concern that as the population grows over time and atopic diseases such as asthma become increasingly prevalent, there would be a noticeable contribution to greenhouse gas emissions and further accelerate the process of climate change. Though there have been attempts to reduce HFC use globally through policy making, such as the 2019 Kigali Agreement [5], more research is required to seek methods of eliminating propellant use in pMDIs. Increased awareness is required of such changes, which need to be communicated to inhaler device stakeholders such as manufacturers and suppliers. Alternatives that have a reduced GWP value but do not have an increased risk of adverse effects in patients can be considered in reducing carbon emissions from propellant sources.

The use of single-use polymer plastics, such as polypropylene, in asthma inhaler devices has been found to play a role in requiring large energy usage with high output waste products, given their disposability. Single-use polymers are most commonly found in the caps used, spacer add-ons, and packaging of the asthma inhaler devices. Given its single-use property, the disposal of plastics mainly occurs through two main methods: incineration through energy-to-waste facilities with energy recovery and landfill disposal. The basis for incineration involves transporting the used inhalers to a facility called Energy-to-Waste, whereby the plastic products are placed into an incinerator and thermally degraded at high temperatures [6]. This process not only degrades the polymer but also releases energy in the process, meaning that the emissions are partially offset. Given the high operating costs and reduced awareness of such a disposal method, it is seldom used. The most common method of disposal is via landfill, given its ease of accessibility and low operating costs. The majority of landfill inhalers consist of propellants that have not been completely used before disposal, hence there is a prolonged period of HFC emissions as inhalers are disposed of in landfills, further contributing to carbon emissions.

Inappropriate prescribing of inhalers, especially with reliever medications such as salbutamol, has increased the number of inhalers used, which would not have been necessary, leading to more plastic consumption and propellant use per device. According to Azzi et al, almost 1/3 of patients seeking short-acting beta agonists (SABA) from community pharmacies have not been prescribed preventer treatment such as inhaled corticosteroids (ICS), even though the treatment is part of current guidelines for maintenance therapy. The use of appropriate maintenance treatment has also been shown to reduce hospitalisation for patients diagnosed with asthma (OR 0.73 (95% CI 0.57-0.94)) [7]. Reduced hospitalisation would lead to reduced reliever therapy used in settings such as the emergency department, and lower cost of resources expended, including beds, personal protective equipment, and utilities, which mitigates potential sources of carbon emissions.

Methods of reducing carbon emissions from inhaler use

One of the most effective methods of reducing carbon emissions is the use of alternative inhaler devices, such as DPI or SMI. An advantage to using the SMI and DPI is that they are both propellant-free, leading to the devices having smaller carbon footprints and GWP values ranging from 357 to 917. Some developed SMI and DPI inhalers release carbon emissions almost 100-200-fold lower than pMDI devices. In one study conducted by Pernigotti et al., the switch from pMDI to DPI/SMI use was predicted to reduce carbon emissions by 64-71%, whereby the main sources of carbon emissions would arise from the manufacturing and disposal process of the inhaler devices [8].

Given that there are patient populations where prescribing SMI/DPI use is not recommended, such as patients under the age of six [9], there have been initiatives set out to reduce propellant emissions from pMDI via research and development of alternative, cleaner propellants. Since the Kigali Amendment to the Montreal Protocol, pharmaceutical companies have been encouraged to manufacture inhalers of low-GWP propellants in pMDI devices, such as HFA-152a or HFO-1234ze. Given that the GWP of HFC 152a is ten times lower than that of HFA-134a, the standardised use of HFA-152a is estimated to reduce carbon emissions of pMDI by upto 92% [10]. Further reductions would be achieved through the use of HFO-1234ze, which has been shown to have a GWP a hundred times lower than HFC-134a.

The use of recycling programs for unwanted components of inhaler devices, such as plastics and metals, would ensure that inhaler devices that end up as landfill waste can be properly treated to reduce propellant leakage. One such program includes the National Return and Disposal of Unwanted Medicines Program (NatRUM). This is a publicly funded program that involves providing disposal bins to community pharmacists for medications that need extra precaution in disposing (not limited to inhaler devices) [11]. In the case of inhaler devices, patients are encouraged to dispose of devices once used at their pharmacy collection site, which then will be collected by specialist disposal services and transported for incineration as per environmental regulation and legislation. Further expansive recycling schemes involve additionally collecting the HFC emitted to be reused in other applications, such as refrigerator coolants and air conditioners. Internationally, the implementation of such a recycling program, such as the Take Action for Inhaler recycling (TAKE AIR) in the United Kingdom, was able to recycle up to 50000 inhalers with a total of 305.3 tons of CO2 saved from entering the atmosphere between its pilot years of 2021-2023 [12]. To ensure the success of such programs, increased community education and awareness of such programs by clinicians and government organisations, as well as providing appropriate funding to recycling programs, is required through consultation with various stakeholders.

Prescribing strategies by clinicians in reducing carbon emissions

Prescribing clinicians hold responsibility not only at the individual patient level to provide the most appropriate evidence-based care, but also to engage in advocating for community health through improving environmental health. The most pertinent method for reducing inhaler-related carbon emissions is through appropriate diagnosis, management, and review of patients with lung disease. Inappropriate diagnosis of obstructive lung diseases such as COPD and asthma has been noted in studies, including the BOLD study [12]. The study recorded patients who were either not diagnosed with obstructive lung disease, whilst scoring a “forced expiratory volume in 1 second” (FEV1) and “forced vital capacity” (FVC) ratio (FEV1/FVC) of <70%, or were diagnosed but did not meet FEV1/FVC criteria. Out of 3355 participants, 233 (6.9%) participants had undiagnosed COPD whilst meeting the FEV1/FVC criteria, whilst 74 (2.2%) participants were diagnosed with COPD but did not meet criteria [13]. Underdiagnosis or misdiagnosis both lead to inappropriate inhaler prescribing, where underdiagnosed patients would be prescribed reliever medications instead of preventer medications, leading to increased hospitalisation and turnover of pMDI use, whilst misdiagnosed patients were prescribed both preventer and reliever medications when they did not require them.

Prescribers have also been encouraged to adhere to routine monitoring and medication reconciliation, especially for patients who have been recently hospitalised, pregnant, or have had poor asthma control using reliever medications at least twice a week within 4 weeks. A study conducted by Azzi et al. reviewed 412 patients, of whom 289 patients (70.1%) reported overuse (use of relievers twice or more in a week over four weeks) of short-acting beta agonist (SABA) medication [6]. A lower proportion of overusing SABA patients were also instructed to use preventer medication compared to non-overusing patients (41% vs 63%, p<0.01). Furthermore, 25.9% of patients reported using their preventer on an “as needed” basis, with 13.3% of patients believing that preventer medications “do not work” [8]. Encouraging use of preventer medication, such as inhaled corticosteroids (ICS), has been reported to reduce the severity and frequency of obstructive airway disease, such as asthma, reduce hospitalisation, and reduce asthma mortality by up to 21% (adjusted ratio 0.79 (95% CI 0.65-0.97)) [14]. From the perspective of carbon emissions, Ng et al. reported greenhouse gas emission estimated at 108-145 kgCO2e per person contributed to salbumatol use compared to 32 kgCO2e per person contributed to ICS use [10]. As prescribing clinicians, it is in the best interest of the patient to ensure that there is proper education and communication regarding preventer medication use to allay any misconceptions and concerns.

Prescribing clinicians are also able to contribute to reducing carbon emissions through further awareness of delivery devices, including SMI and DPI inhalers. As aforementioned, SMI and DPI devices do not require propellants to deliver medication, which prevents the release of greenhouse gases. Multiple systematic reviews, including Brocklebank et al and Twohig et al., have found that SMI/DPI inhalers have similar levels of effectiveness in treating obstructive airway diseases [14,15]. This presents as an attractive alternative to pMDI that patients will prefer to use, given it is environmentally friendly relative to pMDI without the compromise of pharmacological benefit. To further enhance use, education about other environmentally conscious devices to patients, as well as other clinicians and medical students, provides the awareness required for higher uptake of SMI/DPI inhaler devices, hence contributing to reducing carbon emissions.
